# Integrating properties and conditions to predict spray performance of alternative aviation fuel by ANN model

**DOI:** 10.1186/s13068-023-02408-x

**Published:** 2023-11-08

**Authors:** Ziyu Liu, Zimu Tang, Xiaoyi Yang

**Affiliations:** 1 School of Energy and Power Engineering, Energy and Environment International Center, Beihang University, Haidian District, 37 Xueyuan Rd., Beijing, 100191 People’s Republic of China; 2 School of Aeronautic Science and Engineering, Beihang University, Beijing, China

**Keywords:** Alternative fuel, Spray characteristics, Nozzle, Schlieren image, Cone angle, Liquid length

## Abstract

Alternative aviation fuel has been confirmed benefits for GHGs reduction and energy saving. Alternative fuel use should meet drop-in fuel requirement, and one of the important factors to ensure combustion completeness is to achieve spray requirement in the whole envelop of flight. Alternative fuels are characterized different fuel properties at low temperature comparison with traditional jet fuel. For understanding fuel properties and spray-related processes under different conditions, alternative aviation fuel, including Fischer Tropsch (FT), cellulose hydrotreating jet fuel (CHJ) and traditional jet fuel (RP-3), were investigated spray performance. According to empirical equation deduced from experiment data (283 K-343 K), deviations to RP-3 enhanced significantly on surface tension and viscosity at low temperature aera (243 K-273 K). As the complex and discontinuous interaction between nozzle structure and fuel properties with temperature, and thus it is difficult to obtain appropriate empirical equation or simulation results at low temperature. Moreover, non-drop-in fuel like pure FT fuel cannot comply with the same spray mechanism as drop-in fuel. The artificial neural network (ANN) approaches have been involved to solve the complex relationship of properties with spray performance. ANN-spray model coupling with ANN-mass flow can predict not only cone angle and liquid length but also SMD and velocity in liquid zone and droplet zone with above 0.99 total correlation coefficient. Coupling simulation results of mass flow and spray performance, FT and CHJ as well as blend fuels present more obvious difference to RP-3 in droplet size distribution and velocity distribution at low temperature.

**Supplementary Information:**

The online version contains supplementary material available at 10.1186/s13068-023-02408-x.

## Introduction

Alternative aviation fuel has been confirmed benefits for GHGs reduction and energy saving [1, 2]. Aviation fuel is to transfer chemical energy into heat energy in combustor of engine. One of the important factors to ensure combustion completeness is to achieve spray requirement. To meet the criteria for commercial use, alternative aviation fuel should be certified as ‘drop-in’ fuel in spray level, which could avoid engine redesign and fuel system update. Although several alternative fuels have been investigated on spray performance [3, 4], the certifying of alternative aviation fuel on spray level are still challenges due to special property of alternative fuel derived from various feedstock and refining pathway.

Spray performance is characterized with droplet size distribution, spray pattern, and flow field in the combustor, which control combustion completeness [5]. According to spray characteristics of single composition fuel and compound composition [6], fuel physical property is insignificant on the spray characteristics at high injection pressures. No significant difference in vapor penetration rates was found and was mainly controlled by momentum. D. Sivakumar et al. [3] investigated atomization performance of camelina-derived aviation fuel. The droplet size predicted by spray empirical model could agree well with experimental results but the droplet size predicted by theoretical model presented higher than experimentally measurement. The analysis on the comparison of spray measurements between the alternative fuels and Jet A-1 sprays suggests that overall, the primary and secondary atomization characteristics of the jatropha-derived alternative fuels are almost comparable to the Jet A-1 [4]. The spray characteristics of two types of gas to fuel (GTL) blends and conventional Jet A-1 fuels are investigated downstream of a pressure swirl nozzle exit at two injection pressures [7]. The lower viscosity and surface tension of blend fuel led to faster disintegration and dispersion compared to those of Jet A-1 fuel. The difference of radial profiles of droplet size is minor and the axial mean velocities of GTL fuels is higher than Jet A-1 fuel. Fuel physical properties are insignificant on the spray characteristics at high injection pressure and showed same spray characteristics in low pressure for GTL and Jet A-1 [8]. However, the spray performance showed that GTL fuel has higher far field spray cone angle than Jet A-1 [9]. The different alternative drop-in fuel conducts the different sensitivity on spray, which could be attributable to the variation in their fuel properties.

Fischer–tropsch fuel blend with RP-3 conventional jet fuel was investigated spray performance [10]. FT fuel has higher velocity and larger droplet size than RP-3 near the nozzle exit. For further understanding the effects of chemical compositions on spray performance, traditional jet fuel (RP-3) with blend alternative compositions including paraffins, cycloparaffins, aromatics were investigated the spray performance from the view of carbon number distribution and classification distribution in jet fuel compositions [11]. The deviation of fuel properties contributes less on the variations of cone angles. However, the liquid length changed obviously at various pressure. Viscosity and vapor pressure present more sensitive than density and surface tension, which is coincident with that the greater liquid fuel viscosity and surface tension slow the droplet breakup and atomization process [12]. The blend fuels with high deviation in density, viscosity and surface tension cannot be considered as the drop-in jet fuel due to the change of breakup mechanism in spray process. $\mathrm{Lb }(\mathrm{f}) \left[{\sigma }^{0.25} {{\mu }_{f}}^{0.25} {{\rho }_{f}}^{0.25}\right]$ and $\mathrm{Oh}\left(\mathrm{f}\right) [{{{{\mu }_{f}\sigma }^{-0.5}\rho }_{f}}^{-0.5}]$ extracted could be considered to certify the deviation of spray performance compared with RP-3. As the higher specific heat and heat of vaporization will result in a greater amount of heat transfer from the ambient to vaporize, the total thermal energy for heat up and vaporization could influence liquid length of the liquid fuel [13]. The evaporation was caused by high relative velocities between the droplet and the surrounding gas and resulted in a fast evaporation especially light components with higher vapor pressure and lower boiling point in blend fuels. By calibrating through evaporation constants, the empirical equations of droplet size can agree well with the experimental data of blend fuel with light components.

The success of high-altitude re-ignition is closely related to atomization and thus atomization at low temperature is very important for the safe operation of the engine. The sensitivity of fuel properties for spray performance conducted obvious change at different temperature. Alternative fuels are characterized different fuel properties and difference fuel property change with the temperature comparison with traditional jet fuel due to fuel composition variation [14], which remain uncertainty on the effect of fuel property on spray at low temperature.

However, few researches have been investigated on how compositions of alternative fuel blend affect properties and subsequently spray performance at low temperature. Moreover, spray-related processes under low temperature are much more complex, which was considered as the important effects on droplet size. For improving the understanding of fuel properties on spray performance, the properties of jet fuels were investigated for obtaining temperature effects on properties. The spray performance related with the integrating the properties have been assessed quantitatively by artificial neural network (ANN) approaches for achieving the complex interaction of multiparameter. The methodologies of ANN-spray model could predict the spray performance of sustainable alternative jet fuel blend at low temperature.

## Methodology

### Compositions and properties

The compositions of jet fuels were investigated by GC–MS instrument (Agilent 7890A-5975C) with a HP-5 capillary column. The sample was diluted with dichloromethane (1:10, V/V) and the injected volume was 1 μL with 20:1 split ratio. The oven temperature started at 50 °C for 2 min, and then ramped to 175 °C at 5 °C/min for 2 min, and finally ramped to 300 °C at 2 °C/min for 2 min. The injector and detector temperatures were 280 °C and 150 °C. The mass spectrometer scan ranged from m/z 30 to m/z 750, GC–MS spectra charts were in Additional file 1. The chemical substance was captured and identified using NIST 17 with 103 compounds in RP-3, 118 compounds in FT, 127 compounds in CHJ.

### Spray performance measurement

Cone angle and liquid length are investigated by shadow measurement while size and velocity of droplets by Phase Doppler Anemometry (PDA, DANTEC dynamics, model BSA P60). The atomization experiments were conducted at 0.2 MPa–0.7 MPa for RP-3(conventional jet fuel), CHJ (cellulose jet fuel) [15], FT, CHJ 50% with RP-3 and FT 50% with RP-3. The index of refraction and density of sample is RP-3 (1.4366 nD, 0.779 kg/m^3^), CHJ (1.4379 nD, 0.7905 kg/m^3^), FT (1.4170 nD, 0.7445 kg/m^3^), CHJ 50% (1.4373 nD, 0.7848 kg/m^3^) and FT 50% (1.4268 nD, 0.7618 kg/m^3^).

The PDA measuring origin point was set at 1 mm downward from the nozzle exit and the measuring points were carried out from z = 1–40 mm. The measurement points close to the origin are relatively more intensive near the nozzle exit. At each location, the measurement was set to collect either a maximum of 6000 samples or a minimum of 15 s.

**Fig. 1:**
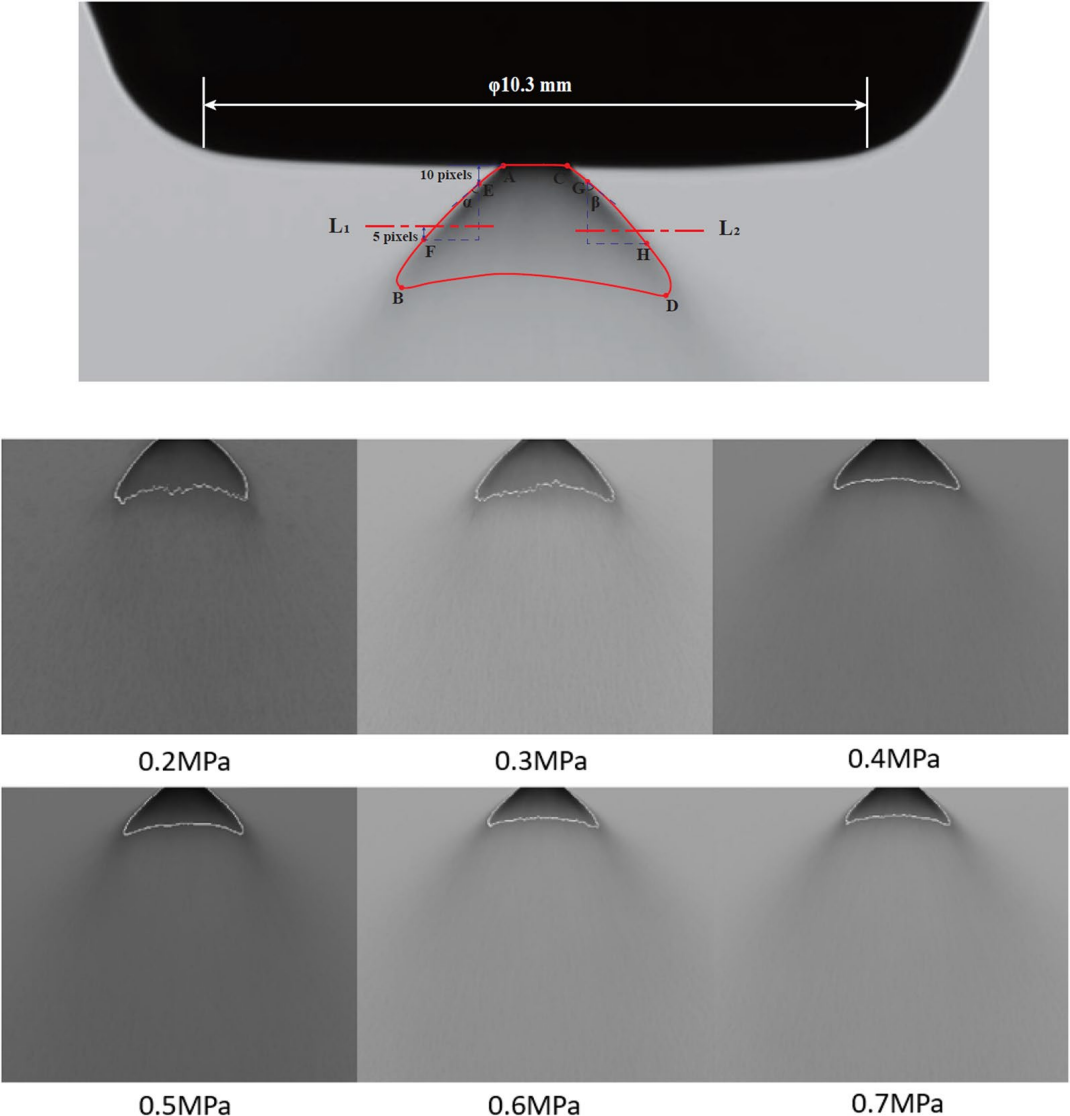
Cone angle and liquid length identification

The shadowgraph system is composed of Xenon LED light (1000LM), concave mirrors (diameter 203 mm, 800 mm focal length), and high-speed cameras. The images with spray information are reflected through the concave mirror on the camera [11]. Each type of fuel in a certain pressure takes more than 20 images combined in a group at the same position and under the same lighting conditions. Each image in the group would convert into a grayscale image and add the pixel value of the corresponding position for distinguishing the boundary of atomization area according to the grey level value, shown in Fig. 1.

The boundary of the spray cone was defined 4 points as Point A, B, C and D. Point E in the line AB and point G in the line CD are both 10 pixels downward from line AC. both of Line 1 and line 2 are at half position of the vertical distance of Point A–B and Point C–D, respectively. Point F and Point H is 5 pixels downward from Line 1 and 2. Line EF with vertical line formed angle αwhile line GH with vertical line formed angleβ. Spray cone angle equals to the sum of α and β. The liquid length is the average vertical distance of every pixel point in arc BD to Line AC, which is calibrated by the diameter of the nozzle bottom width (10.3 mm).

### ANN-spray model computation framework

**Fig. 2:**
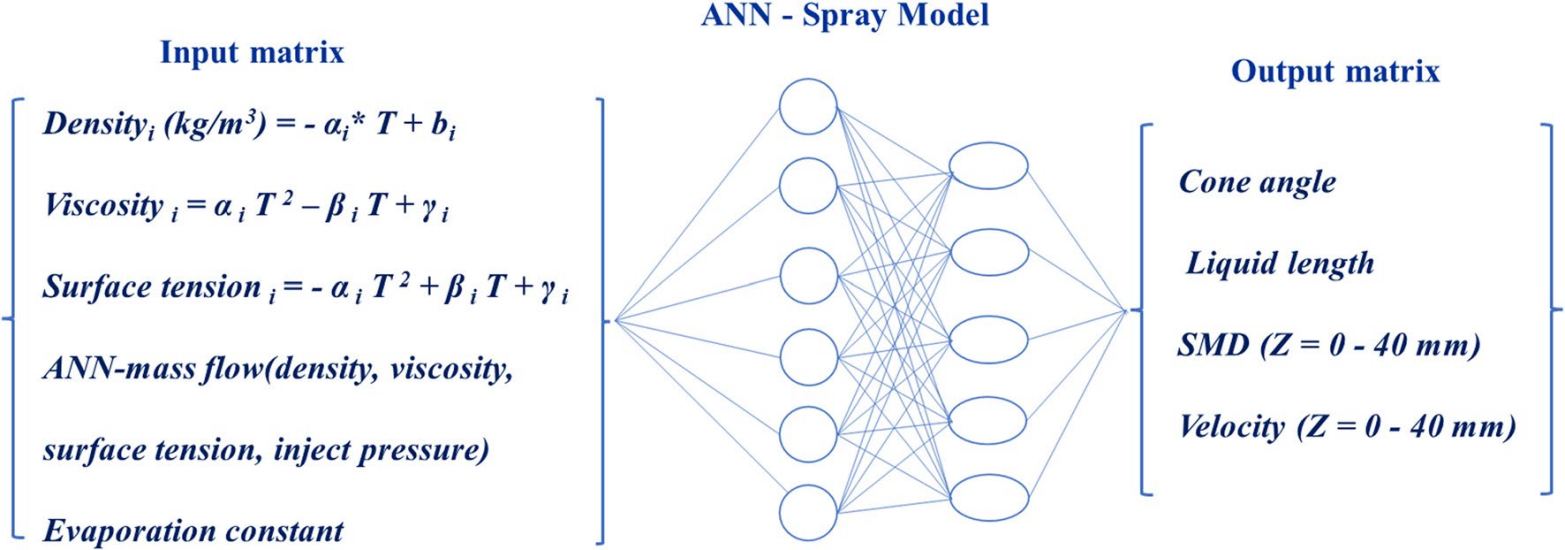
ANN-spray model logical diagram

ANN-spray model was trained by experiment data at 293 K. The critical parameters were extracted based on test data and theory analysis (in Spray performance simulation at low temperature by ANN-spray model). As mass flow should be involved in ANN-spray model computation framework as input parameter, which is related with viscosity, density, and interaction of nozzle structure with operation condition, environment condition, and fuel property. ANN-mass flow model should be established in advance based on test data and theory analysis (in ANN-Mass flow model). For ANN-mass flow model, input matrix includes pressure, viscosity, density, and surface tension and output matrix is mass flow. For ANN-spray model, input matrix includes operation condition (pressure and mass flow), environment condition (temperature), and fuel property (viscosity, density, surface tension, and vapor pressure). ANN-spray model logical relationship is given in Fig. 2.

For achieving the appropriate neural network modelling, 70% of data set is randomly selected as the training set and 15% as test set and other 15% as for validation. Levenberg–Marquardt algorithm was chosen in the process of training neural networks. ANN-spray model obtained was reinstalled input matrix data at 273 K. Pressures are set in the range of 0.3–0.7Mpa and viscosity, density, surface tension at 273 K is derived from empirical equations (in Compositions and properties with temperature effects) while mass flow is calculated based on ANN-mass model. The output matrix includes cone angle, liquid length, SMD (Z = 0–40 mm) and velocity (Z = 0–40 mm).

## Results and discussion

### Compositions and properties with temperature effects

**Fig. 3:**
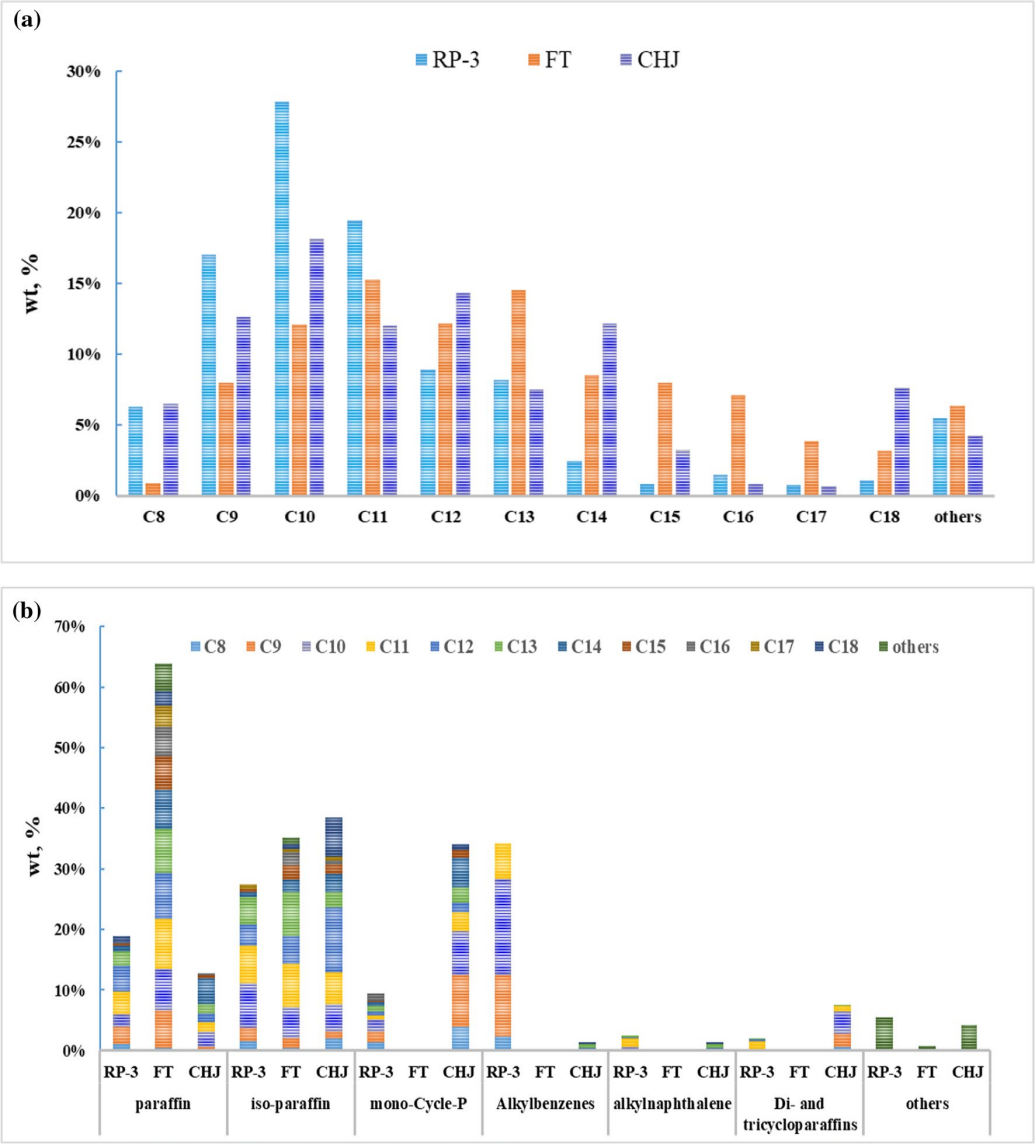
Carbon number distribution and classification of RP-3, FT, CHJ. **a** Carbon number distribution; **b** Classification

The compositions have been compared in carbon number distribution and classification distribution. For complying with boiling point requirement of drop-in fuel, the carbon number distribution of all fuels mainly displays in the range of C8 to C18. RP-3 is characterized a normal distribution from C8 to C18 centered on C10 and C11 while FT is also characterized a normal distribution centered on C10, C11, C12. Although CHJ carbon number distribution is characterized almost a normal distribution centered on C10, C11, C12 in the range of C8–C16, C18 content in CHJ reached at 7.65%. From the view of classification, RP-3 is characterized with high alkylbenzenes while CHJ is characterized with high cycloparaffin. FT is mainly composed of n-paraffin (63.8%) and iso-paraffin (35.2%), given in Fig. 3.

**Fig. 4:**
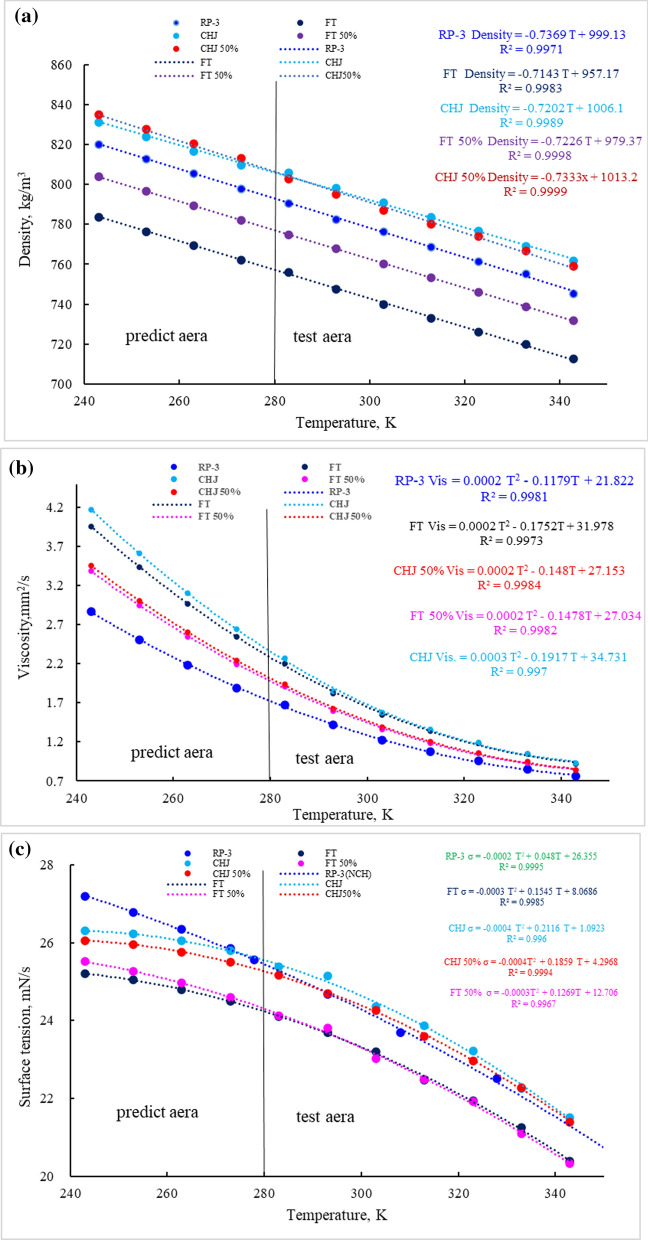
The relationship of properties with temperature. **a** density; **b** viscosity; **c** surface tension

The composition change in carbon distribution or classification could result in the fuel property change, and environment temperature change in the flight envelope also leads to fuel property change. The key properties of jet fuels for spray performance, including density, viscosity, surface tension, were investigated relationships with temperature, given in Fig. 4.

Fuel density as a function of temperature is given in Fig. 4a. At the same carbon number, the density usually follows the order as aromatics > cycloparaffin > n-paraffin > iso-paraffin. At the same temperature, the density of RP-3 is higher than FT (higher paraffine content) but lower than CHJ (higher cycloparaffin content). The density of CHJ 50% blend is quite as same as those of CHJ while the density of FT 50% blend is close to weighted average of FT and RP-3. These phenomena indicated that the total volume of blend fuels is lower than the sum of individual volume of fuels, which could be attributed to the small molecules into spaces among molecules. The densities of all fuel decrease with temperature and comply with linear relationship with temperature (*Density (kg/m*^*3*^*)* =− α* T (K) + *b*). The slopes of downward trend with temperature are in the range of 0.714–0.737, which could be attributed to condensation effects.

Fuel viscosity as a function of temperature is given in Fig. 4b. Viscosity reflects the friction force among molecules in the fluid, which is closely related to the size and structure of molecules. At the same carbon number, the viscosity usually follows the order as cycloparaffin > aromatics > n-paraffin > iso-paraffin. Viscosity of the larger molecular weight of the hydrocarbon usually shows higher viscosity. For a given carbon number, naphthene generally has slightly higher viscosity than paraffins, alkylbenzene and even cycloparaffin. The viscosity with the ring structure is usually greater than that with the chain structure, and the molecules without side chain have greater viscosity than with side chain. For the relationship of viscosity with temperature, temperature has great influence on the viscosity of fuel, and viscosity decreases rapidly with the increase of fuel temperature. According to obtained empirical equation $(Viscosity \, = \, \alpha \, T\left( K \right)^2 -- \, \beta \, T\left( K \right) \, + \, \gamma )$, the relative deviation of alternative fuel and blend fuel to RP-3 present obvious changes at the low temperature. At the range of test area, the viscosities of 50% blend fuels are both close to weighted average of individual fuels.

Fuel surface tension as a function of temperature is given in Fig. 4c. At the same carbon number, the surface tension usually follows the order as same as density, aromatics > cycloparaffin > n-paraffin > iso-paraffin. From the obtained empirical equation $(Viscosity \, = \, \alpha \, T\left( K \right)^2 -- \, \beta \, T\left( K \right) \, + \, \gamma )$, temperature has great influence on surface tension, and curves indicated that RP-3 comply with different rule compared with alternative fuel and blend fuel. As same as change extent of viscosity, the relative deviation of alternative fuel and blend fuel to RP-3 present obvious changes at the low temperature.

For predicting fuel properties at low temperature, density, viscosity, and surface tension of all fuels have been evaluated based on empirical equation deduced from experiment data with all correlation coefficient above 99%, given in Table 1.

**Table 1:** Empirical equation for fuel properties at low temperature, density, viscosity, and surface tension of different type of fuels

	Density = Function (Temp.)	Correlation coefficient
RP-3	*ρ* = −0.7369 T + 999.13	*R*^2^ = 0.9971
FT	*ρ* = −0.7143 T + 957.17	*R*^2^ = 0.9983
CHJ	*ρ* = −0.7202 T + 1006.1	*R*^2^ = 0.9989
FT 50%	*ρ* = −0.7226 T + 979.37	*R*^2^ = 0.9998
CHJ 50%	*ρ* = −0.7333x + 1013.2	*R*^2^ = 0.9999
Viscosity = Function (Temp.)		
RP-3	*μ *= 0.0002 T^2^–0.1179 T + 21.822	*R*^2^ = 0.9981
FT	*μ* = 0.0002 T^2^–0.1752 T + 31.978	*R*^2^ = 0.9973
CHJ	*μ* = 0.0003 T^2^–0.1917 T + 34.731	*R*^2^ = 0.9970
FT 50%	*μ* = 0.0002 T^2^–0.1478 T + 27.034	*R*^2^ = 0.9982
CHJ 50%	*μ* = 0.0002 T^2^–0.148 T + 27.153	*R*^2^ = 0.9984
Surface tension = Function (Temp.)		
RP-3	*σ* = −0.0002 T^2^ + 0.048 T + 26.355	*R*^2^ = 0.9995
FT	*σ* = −0.0003 T^2^ + 0.1545 T + 8.0686	*R*^2^ = 0.9985
CHJ	*σ* = −0.0004 T^2^ + 0.2116 T + 1.0923	*R*^2^ = 0.996
FT 50%	*σ* = −0.0003T^2^ + 0.1269 T + 12.706	*R*^2^ = 0.9967
CHJ 50%	*σ* = −0.0004T^2^ + 0.1859 T + 4.2968	*R*^2^ = 0.9994

In predict area of low temperature (243 K–273 K), there are more obvious deviations to RP-3 both of surface tension and viscosity than in test area (283 K −343 K), which indicated the spray performance of alternative fuels may differ from RP-3 at low temperature

### ANN-Mass flow model

For swirl nozzle based on energy conservation, the mass flow can be simplified as:

$${\dot{m}}_{f}= [\mathrm{nozzle\, structure}] [\mathrm{ fuel }\sqrt{{\rho }_{l}} ] [\mathrm{operation }\sqrt{\Delta {P}_{l}}]$$

The mass flow is mainly influenced by the drop of injection pressure ($\Delta {P}_{l,}$) and density (${\rho }_{l}$). For jet fuel as viscous fluid, the mass flow can be expressed by coupling with the nozzle geometrical parameter, flow conditions and fuel properties, as follows:

$$\dot{m}_f = C_d A_n \sqrt {2\rho_l \Delta P_l } \, = \,[{\text{nozzle structure }}\& {\text{ fuel}}\left] \, \right[{\text{ fuel}}\,\sqrt {\rho_l } \,\left] \, \right[{\text{operation}}\,\sqrt {\Delta P_l } ]\, = \,f\left( {{\text{nozzle}},\rho_l , \mu_{f,} \Delta P_{l,} } \right)$$

**Fig. 5:**
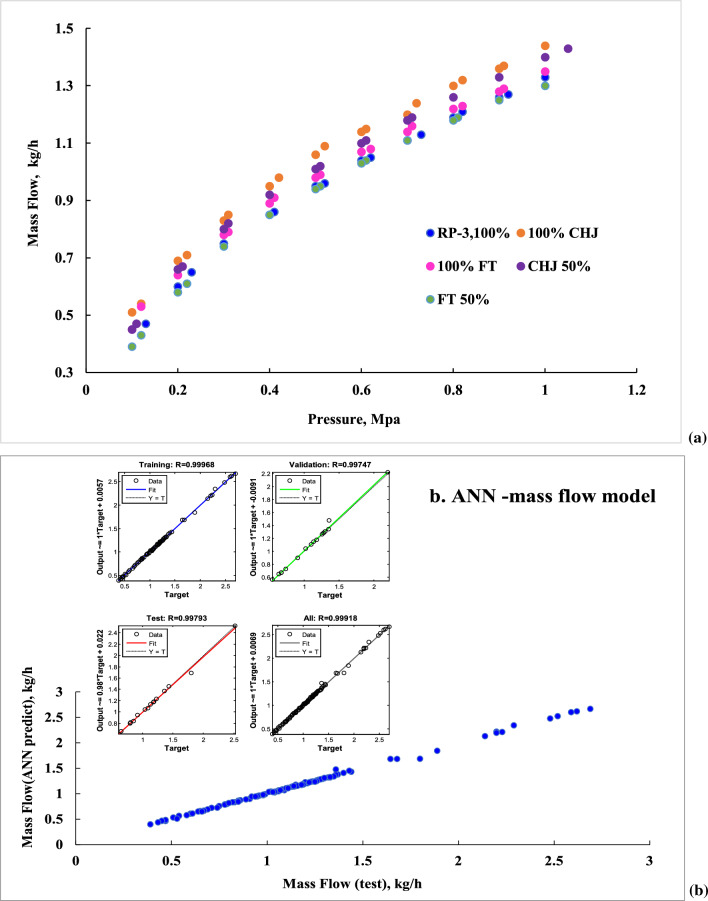
Mass flow at various pressure. **a** test; **b** ANN-mass flow model (training 0.99968; validation0.99747; test 0.99793; all 0.99918)

The interaction of jet fuel with nozzle structure should be involved in the mass flow. As the complex interaction between nozzle structure and fuel properties could lead to the change of relationship of nozzle structure with the change extent of fuel properties, and it is difficult to obtain appropriate empirical equation solution or theory equation solution. Moreover, even though the appropriate empirical equation or theory equation can be deduced from the test data, it cannot apply to predict the mass flow at low temperature. Therefore, artificial neural network (ANN) has been involved to achieve the challenge mass flow prediction at low temperature. The test mass flows at 293 K, given in Fig. 5a, present increase with injection pressure. According to Fig. 5b, ANN-mass flow model obtained meets the experiment results well with high accuracy, which indicated that ANN can achieve to establish the complex relationship of structure with fuel properties.

### Spray cone angle and liquid length

The deviations of alternative fuel blends compared with RP-3 jet fuel presented multiformity in both of cone angle and liquid length, which could be induced by property variation.

**Fig. 6:**
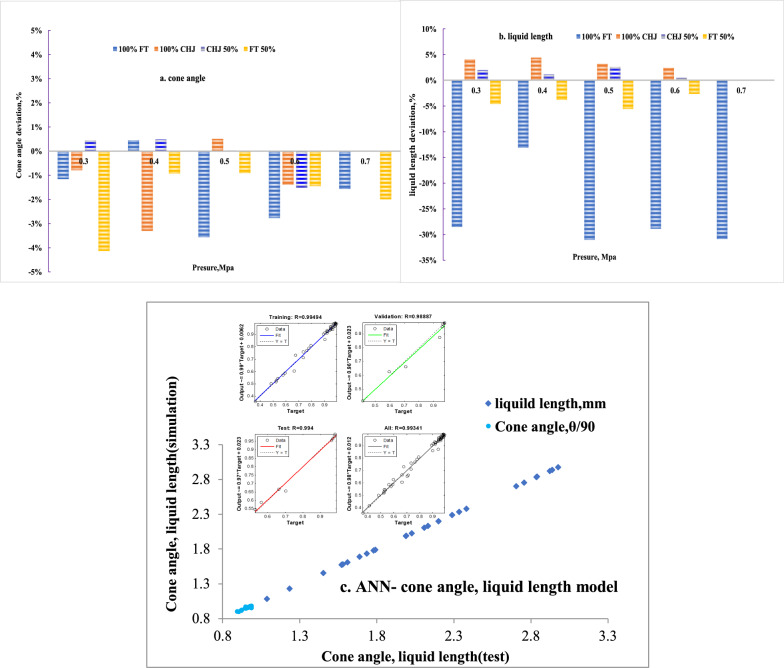
Alternative fuel and blend fuel in comparison with RP-3. **a** cone angle; **b** liquid length; **c** ANN- cone angle, liquid length model

For cone angle, all deviations to RP-3 are in the range of ± 5% at various pressure, which comply with the characteristics of swirl nozzle, given in Fig.6a. For liquid length, FT appears the significant negative deviation while FT 50% appears the less negative deviation. CHJ and CHJ 50% both appears the positive deviation within 5%, given in Fig.6b. CHJ blend and FT blend present the different deviation to RP-3, which should comply with the deviations of properties. Cone angle is influenced by fuel properties, including density, viscosity and the structure of swirl atomizer [11, 16]. Further, cone angle can be integrated as:

$${\text{Cos}}(\theta /{2}) = \, [{\text{structure }}({\text{nozzle}} ][{\text{operation}}\left( {{\Delta P}_{\text{f}} } \right)][{\text{fuel }}\left( {\mu_f ,\rho_f } \right)]$$

According to previous research [11, 16, 17], the liquid length of blend fuel can be integrated as:

$$L_b \, = \,[{\text{structure }}({\text{nozzle}} ][{\text{operation}}\,\left( {{\Delta P}_{\text{f}} } \right)][{\text{fuel}}\,\left( {{\upsigma },\mu_f ,\rho_f } \right)]$$

The interaction among structure, operation and fuel can influence the spray performance coupling with the type of nozzle, which are all difficult to quantify as same as mass flow. Specially, non-drop-in fuel cannot comply with the same spray mechanism as drop-in fuel [11], and this conclusion confirmed that complex interaction could change with fuel property. The density of pure FT fuel is below 775 kg/m^3^ at 298K, which cannot comply with drop-in fuel in the range of 775–840 kg/m^3^. In Fig.6b, the higher deviations of 100% FT were observed. Therefore, ANN model has been involved for achieving the complex relationship of cone angle and liquid length with structure, operation, environment, and fuel as well as the interaction among them. Input matrix includes operation condition (mass flow, pressure), environment condition (temperature) and fuel property (viscosity, density, surface tension). The output matrix includes cone angle and liquid length. The connection weights in the hidden layer were optimized iteratively by the back-propagation algorithm with Levenberg–Marquardt rule to minimize mean square error between test data and predicted outputs. In the domain of neural network modelling by multi-round iteration, ANN-cone angle and liquid length model with high-precision was created with total 0.993 correlation coefficient (training data at 0.995 correlation coefficient, validation at 0.989 correlation coefficient, and test at 0.994 correlation coefficient), given in Fig. 6c.

### Droplet size and velocity

The droplet size and velocity distribution control flame profile, which are analyzed at the liquid sheet zone and the droplet zone, respectively.

**Fig. 7:**
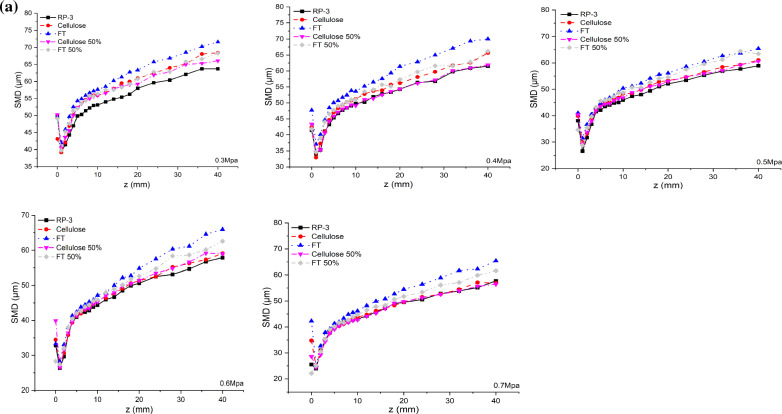

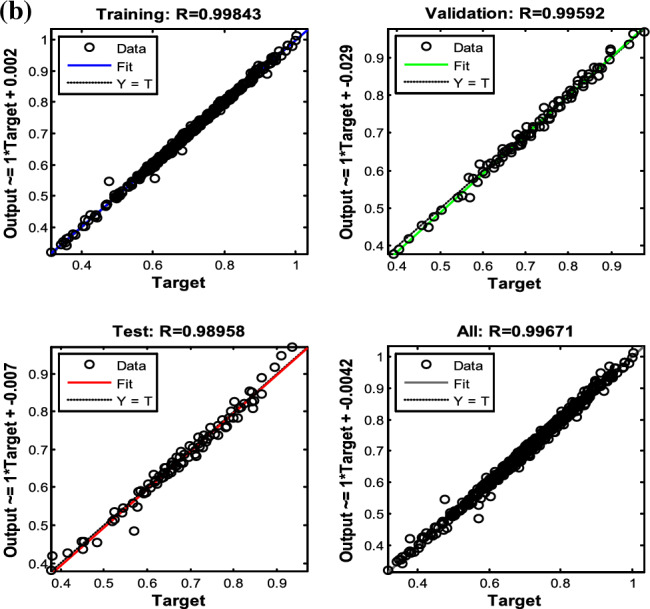
SMD downstream of the nozzle exit at various inject pressure. **a** average axial SMD; **b** ANN- SMD model

In the liquid sheet zone, droplets splashed from liquid sheet by aerodynamic force, which present decrease with further location from the atomizer exit. Axial SMD appears obvious different at z < 3 mm (liquid sheet zone) but quite similar at 3 mm < Z < 5 mm (junction area between liquid zone and droplet zone), given in Fig. 7a. The obvious effects of blend fuels on liquid sheets presented near the nozzle despite of at low pressure or at high pressure. In the range of 5 < Z < 10 mm, the significant different only present at low pressure condition while in the range of Z > 10 mm, detectable different of SMD can be captured at the various pressure condition. In the droplet zone at further locations from the atomizer exit, droplets appear a significant rise in size, which could be attributed to the significant difference of blend fuel on the combined effects of droplets evaporation and droplet-to-droplet collisions. SMD prediction presents more complex than cone angle and liquid length due to more interaction involved. According to ANN-SMD model obtained, the total correlation coefficient can get to 0.9967 given in Fig. 7b.

**Fig. 8:**
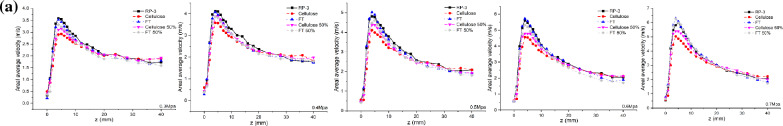

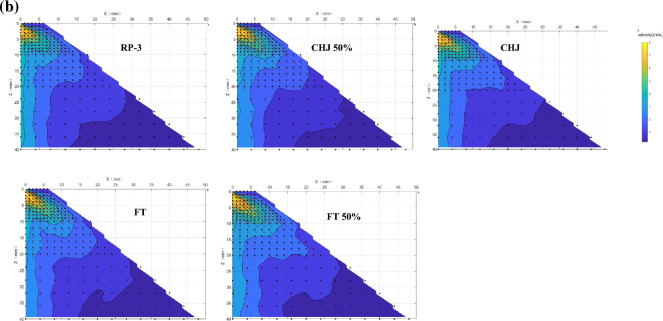

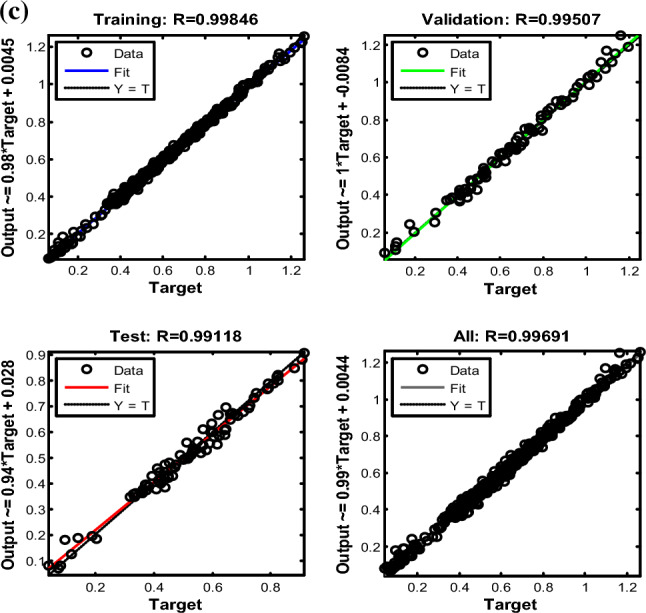
Velocity of various fuels. **a** Mean axial velocity at various inject pressure; **b** velocity contour at 0.5 MPa injection pressure; **c** ANN- velocity model

The axial velocity distribution downstream of the nozzle exit at various inject pressures are shown in Fig. 8a. In the liquid sheet zone, the droplet splashed from liquid sheet was unstable and stochastic with lower droplet velocities, the statistical data of the mean axial velocities present the decrease with further locations from the atomizer exit. After the liquid sheet breaks into droplets, the initial droplets with the highest mean axial velocity gradually drop with further leave. The bigger droplets with higher momentum can reach to periphery region while small droplets with lower velocity appeared at intermediate region. The obvious variation of different fuel presents at the position close to the nozzle exit as SMD profile. In recirculation zone, all fuels present a backflow area with highest concentration near the nozzle exit but with different profile, given in Fig. 8b. In droplet zone, the droplet velocity of various fuels present significant difference at the range of 3 < Z < 15 mm. FT and FT 50% had similar droplet velocity as RP-3 while CHJ and CHJ 50% present lower droplet velocity at various pressure. For ANN-velocity model, the total correlation coefficient was 0.9969, given in Fig. 8c.

### Spray performance simulation at low temperature by ANN-spray model

The spray field was classified into the liquid sheet zone (primary spray zone) and the droplet zone (secondary spray zone). In the liquid sheet zone (primary spray zone), fuels were accelerated to rotate at high speed in the nozzle by pressure drop, and form a liquid film in the convergent cavity. The liquid film appears continuously at the nozzle outlet and was sheared and broken by the aerodynamic force along the downstream of the nozzle. Weber number is characterized the ratio of aerodynamic force to surface tension, which is defined the type of broken mechanism, and can be expressed by integrating fuel effect on nozzle structure, as follows:

$$We =[structure(\mu \left(T\right),\rho \left(T\right))]\left[operation\left(\Delta p\right)\right][fuel(\sigma \left(T\right), \mu \left(T\right),\rho \left(T\right))]$$

Ohnesorge number is defined as $\mathrm{Oh}=\frac{{\mu }_{f}}{\sqrt{{\rho }_{\sigma d}}}=\frac{\sqrt{We}}{Re}= \frac{Viscous\, force}{\sqrt{inertia\, force*surface\, tension}}$, which indicated contrast of viscous force, inertia force, and surface tension. Along with temperature change, viscous and surface tension could change with temperature while inertia force could change with density.

The influence of temperature is complex and nonlinear on fuel properties and subsequently the influence of fuel properties on spray performance is complex and discontinues. Moreover, interaction between nozzle structure and fuel properties with temperature is also discontinuous, and thus it is difficult to obtain appropriate empirical equation or simulation results at low temperature due to broken mechanism change with fuel properties change.

In the droplet zone (secondary spray zone), *SMDt* of droplet size at t time can be expressed by SMD_0_ of droplet size at initial time based on evaporation law, as follows:

$${SMD}_{0}^{2}-{SMD}_{t}^{2}={K}_{evaporation}$$

The evaporation constant Kevaporation was caused by high relative velocities between the droplet and the surrounding gas and resulted in a fast evaporation especially light components with higher vapor pressure and lower boiling point in blending fuels, which can be defined as follows:

$${\text{K}}_{{\text{evapration}}} = {285}.{\text{95ln }}\left( {{\text{T}}_{{\text{bp1}}0\% } } \right) \, + { 38}.{381}$$

where T_bp10%_ is the temperature of 10% distillation, which indicated that evaporability of fuel cannot ignore in the droplet zone.

**Fig. 9:**
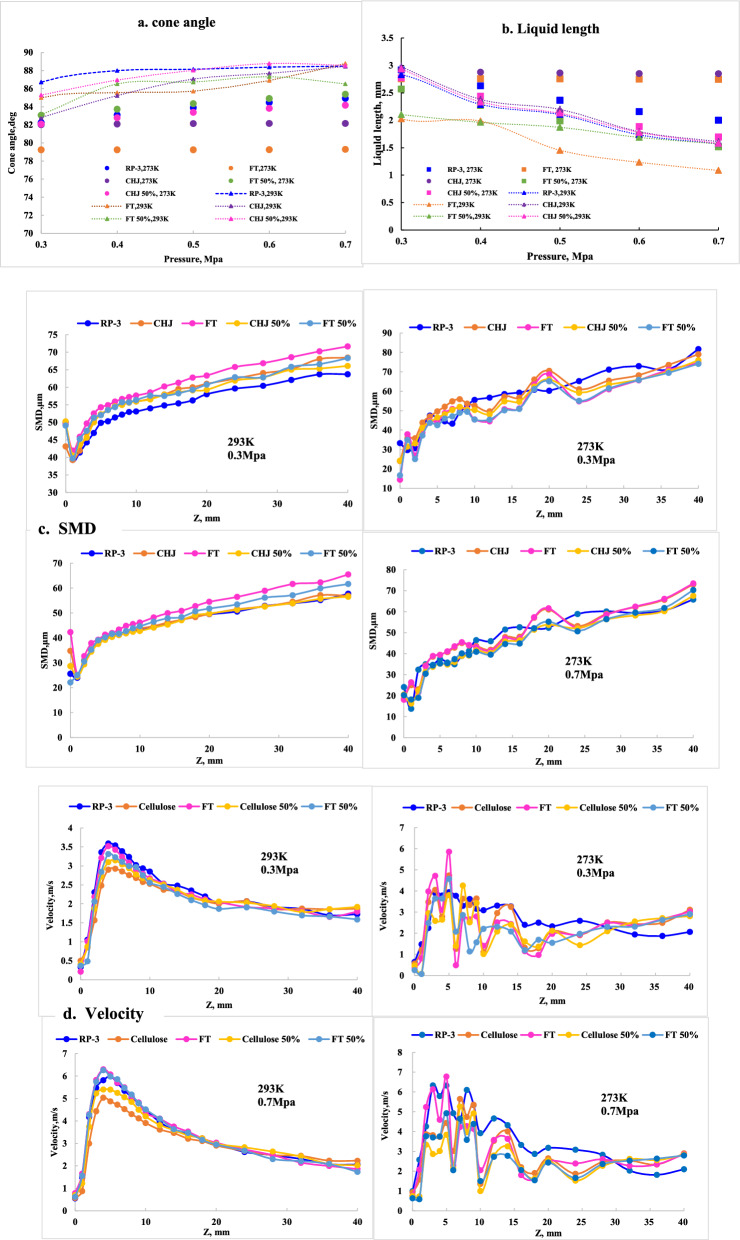
Spray performance at 273K. **a** cone angle; **b** liquid length; **c** SMD; **d** velocity

According to above theory analysis and experimental data analysis, spray performance is related with integrating nozzle structure factors, environment factors, fuel factors, and interaction factors among nozzle structure, operation condition, environment condition, and fuel property. Therefore, for achieving complex interaction of multiparameter, spray performance could be assessed quantitatively by artificial neural network (ANN) approaches. The critical parameters should be extracted and classified by uncertainty analysis. Input matrix of ANN-spray model should compose all factors which could influence spray performance, including nozzle structure factors, environment factors, fuel factors, and interaction factors among nozzle structure, operation condition, environment condition, and fuel property. Although nozzle structure factors can be ignored due to no change of nozzle structure, interaction factors, including nozzle structure with operation condition, environment condition, and fuel property, should be involved in computation framework. The appropriate structures and topology of networks make an important role for achieving ANN performance. The input matrix includes operation condition (pressure and mass flow), environment condition (temperature), and fuel property (viscosity, density, surface tension, and evaporation constant).The simulation results at 273 K are given in Fig. 9 a,b,c,d.

All fuel present obvious change between 273 and 293 K. Cone angles of all fuel decrease at 273 k compared with 293 K. FT and CHJ show the most dramatic decline. Moreover, FT and CHJ appear less increase in mass flow with pressure enhancement, which could be attributed to the influence of high viscosity and poor flowability at low temperature. Liquid length of all fuel increases at 273 K compared with 293 K. As same as cone angles, FT and CHJ show the most change, which indicated that broken mechanism changed due to fuel property change and mass flow change.

From the view of SMD, the liquid sheet zone extended with temperature decrease and larger droplets were simulated in spite of at 0.3 Mpa or 0.7 Mpa. At 273 K, SMD multipeak were formed in the droplet zone and the sizes of droplets enlarged, which is coincident with atomization theory of broken mechanism that surface tension increases with temperature decrease. From the view of velocity, as SMD at 273 K, velocity multi-peak was formed in droplet zone, which presents randomness. In comparison with RP-3, FT and CHJ as well as blend fuels present obvious difference in droplet size distribution and velocity distribution.

## Conclusion

The key properties of alternative fuels for spray performance, including density, viscosity, surface tension, have been investigated relationships with temperature. Fuel properties at low temperature (243–273 K) have been evaluated based on empirical equation deduced from experiment data (283 K −343 K). The deviations to RP-3 increase with temperature further decrease on surface tension and viscosity.

The complex interaction between nozzle structure and fuel properties, the change extent of fuel properties could lead to the change of relationship of nozzle structure with fuel properties, artificial neural network (ANN) has been involved to achieve the challenge of complex interaction and certification of drop-in fuel.

ANN-spray model derived from experiment data was established to simulate the spray performance of fuels at low temperature including cone angle, liquid length, SMD (Z = 0–40 mm) and velocity (Z = 0–40 mm).

### Supplementary Information

**Additional file 1. Fig. S1.** GC-MS spectra charts of RP-3, FT, and CHJ.

## Data Availability

The datasets generated or analyzed during this study are available from the corresponding author on reasonable request.

## Abbreviations

RP-3: Traditional jet fuel
$\dot{m}$: Mass flow rate of liquid
∆P_f_: Injection pressure
$$\theta$$: Spray angle
GTL: Gas to liquid
$$Oh$$: Ohnesorge number
FT: Fischer–tropsch aviation fuel
Oh _(f)_: Fuel characteristics constant of Ohnesorge number
CHJ: Hydrotreated jet fuel
L_b_: Liquid length
PDA: Phase Doppler Anemometry
L_b (f)_: Fuel characteristics constant of Liquid length
SMD: Sauter Mean Diameter
$\upsigma$: Liquid surface tension
${\uprho }_{\mathrm{f}}$: Density of the fuel
${\mu }_{f}$: Fuel dynamic viscosity
