# Supplementary material for: Integrating properties and conditions to predict spray performance of alternative aviation fuel by ANN model
Source: Biotechnol Biofuels Bioprod. 2023 Nov 8;16:171. doi: 10.1186/s13068-023-02408-x (PMC10634133; doi:10.1186/s13068-023-02408-x)
Supplement: Supplementary file 1 — Additional file 1. Fig. S1. GC-MS spectra charts of RP-3, FT, and CHJ. [file 13068_2023_2408_MOESM1_ESM.docx]

**Supplement material**

**Integrating properties and conditions to predict spray performance of alternative aviation fuel by ANN model**

Fig. S1 GC-MS spectra charts of RP-3, FT, and CHJ.
